# Exploring the Biological and Mechanical Properties of Abdominal Aortic Aneurysms Using USPIO MRI and Peak Tissue Stress: A Combined Clinical and Finite Element Study

**DOI:** 10.1007/s12265-017-9766-9

**Published:** 2017-08-14

**Authors:** Noel Conlisk, Rachael O. Forsythe, Lyam Hollis, Barry J. Doyle, Olivia M.B. McBride, Jennifer M.J. Robson, Chengjia Wang, Calum D. Gray, Scott I.K. Semple, Tom MacGillivray, Edwin J.R. van Beek, David E. Newby, Peter R. Hoskins

**Affiliations:** 1 Centre for Cardiovascular Science, The University of Edinburgh, Edinburgh, UK; 2 School of Clinical Sciences, The University of Edinburgh, Edinburgh, UK; 3 Institute for Bioengineering, The University of Edinburgh, Faraday Building, The King’s Buildings, Mayfield Road, Edinburgh, EH9 3JL UK; 4 Clinical Research Imaging Centre, The University of Edinburgh, Edinburgh, UK; 5 Vascular Engineering Laboratory, Harry Perkins Institute of Medical Research, Perth, Australia; 6 School of Mechanical and Chemical Engineering, The University of Western Australia, Perth, Australia; 7 Centre for Clinical Brain Sciences, The University of Edinburgh, Edinburgh, UK

**Keywords:** Abdominal aortic aneurysms, Finite element analysis, USPIO uptake, MRI, Patient-specific modelling

## Abstract

Inflammation detected through the uptake of ultrasmall superparamagnetic particles of iron oxide (USPIO) on magnetic resonance imaging (MRI) and finite element (FE) modelling of tissue stress both hold potential in the assessment of abdominal aortic aneurysm (AAA) rupture risk. This study aimed to examine the spatial relationship between these two biomarkers. Patients (*n* = 50) > 40 years with AAA maximum diameters > = 40 mm underwent USPIO-enhanced MRI and computed tomography angiogram (CTA). USPIO uptake was compared with wall stress predictions from CTA-based patient-specific FE models of each aneurysm. Elevated stress was commonly observed in areas vulnerable to rupture (e.g. posterior wall and shoulder). Only 16% of aneurysms exhibited co-localisation of elevated stress and mural USPIO enhancement. Globally, no correlation was observed between stress and other measures of USPIO uptake (i.e. mean or peak). It is suggested that cellular inflammation and stress may represent different but complimentary aspects of AAA disease progression.

**Electronic supplementary material:**

The online version of this article (doi:10.1007/s12265-017-9766-9) contains supplementary material, which is available to authorized users.

## Introduction

Each year, over 10,000 deaths in the UK are attributed to rupture of abdominal aortic aneurysms (AAAs) [1]. It is thought that AAA rupture occurs when wall stress exceeds wall strength, and this is influenced by a number of biological and mechanical factors [2]. However, the exact mechanisms of AAA rupture are unknown. Various pathobiological processes contributing to AAA development and disease progression have been identified, including infiltration of inflammatory cells such as macrophages, proteolytic degradation of the extracellular matrix (ECM), and neovascularisation. All of these biological processes lead to changes in the mechanical properties of the artery wall including loss of elastin and deposition of collagen that compromises the strength and elasticity of the vessel [3, 4].

It is clear that growth and ultimately rupture of AAAs over time occur as a result of complex mechano-biological interactions within the diseased arterial wall [5]. However, it is unclear to what extent these biological and mechanical changes may co-exist and, indeed, contribute to the propagation of each other. Novel imaging studies have been undertaken in an attempt to understand the biological activity of AAA disease, such as the application of ^18^F-fluoride to detect necrotic inflammation [6] and microcalcification [7] or the application of ultrasmall superparamagnetic particles of iron oxide (USPIO) to detect inflammation [8, 9]. Also, we have previously demonstrated that USPIO can identify areas of mural AAA inflammation, which is associated with more rapid aneurysm expansion [8].

Biomechanical studies can assess properties of AAA, such as wall stress and strength [10], and it has been shown that computational models based on the finite element (FE) method can identify rapidly expanding AAAs [11], assess rupture risk [12], and in some instances may even predict the location of rupture [13, 14].

It has been suggested that focal areas of inflammation may be co-located with focal areas of increased mechanical stress [15]. The aim of this study was to explore the spatial relationship between areas of mural cellular inflammation measured by USPIO uptake on MRI and regions of high tissue stress determined through patient-specific FE modelling, for a group of 50 patients under surveillance for AAA disease.

## Methods

### Study Design and Setting

**Fig. 1:**
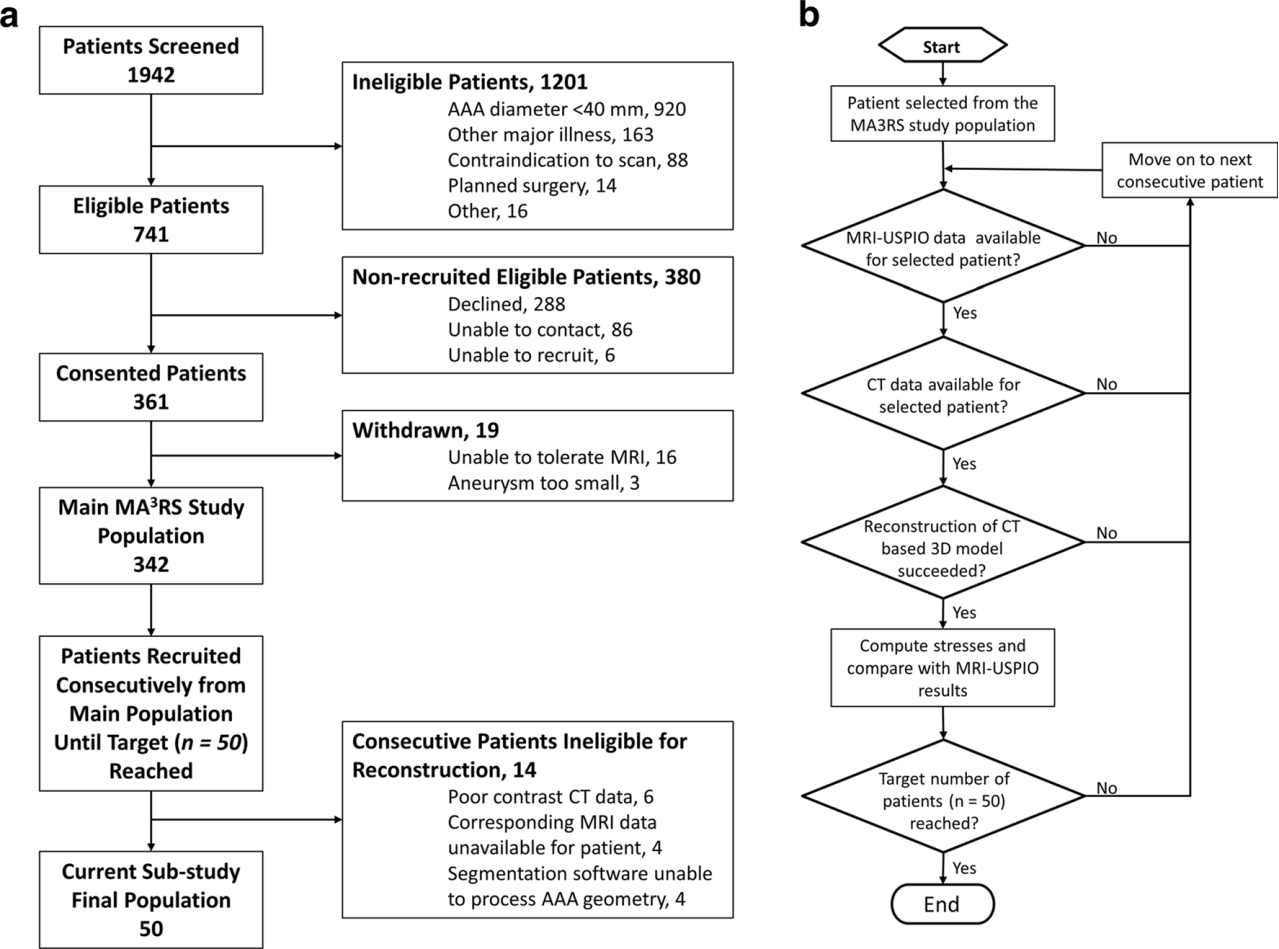
Flowcharts detailing (**a**) patient inclusion/exclusion criteria and (**b**) the patient selection algorithm

This is a sub-study of the MRI in AAA to predict Rupture or Surgery (MA^3^RS) study (http://www.isrctn.com/ISRCTN76413758): a large multi-centre prospective observational cohort study aiming to determine the added value of USPIO-enhanced MRI in predicting AAA rupture or surgery in 342 patients with AAA under routine clinical surveillance. The MA^3^RS study protocol has been described in full elsewhere [16], and the study is in the follow-up stages, due to report in 2017. In brief, the MA^3^RS study cohort underwent USPIO MRI and CTA between November 2012 and December 2014 at the University of Edinburgh, Scotland. Patients were eligible for inclusion if they had an AAA ≥ 40 mm as measured on an ultrasound scan, if they were over 40 years of age, and with no contraindications to USPIO MRI or CTA. Patients were excluded if the suspected aetiology of the aneurysm was inflammatory. An outline of the patient selection criteria is given in Fig. 1a.

For the present sub-study, we selected and analysed scans of consecutive patients recruited into the MA^3^RS study using the process outlined in Fig. 1b. During this phase, 14 of the available consecutive scans were unable to be processed due to factors such as poor contrast on the CT scan (*n* = 6), segmentation difficulties (*n* = 4), and a lack of corresponding MRI-USPIO data (*n* = 4). These patients were therefore excluded from the final analysis. As per the selection algorithm (Fig. 1b), if a patient became ineligible, reconstruction and analysis moved on to the next consecutive patient until the predetermined target sample size of 50 eligible patients was reached. During the selection and reconstruction phase of the study, we were blinded to all demographic data, clinical outcomes, and USPIO groupings for each patient.

### USPIO Magnetic Resonance Imaging and Image Analysis

The USPIO MRI and image analysis techniques used in the study have previously been described [16]. Briefly, participants underwent T2 and T2*-weighted MRI scanning at 3 T (Siemens Magnetom Verio, Erlangen, Germany) both before and 24–36 h after administration of a weight-adjusted dose of USPIO. Both datasets (pre- and post-USPIO) were then registered and analysed, using bespoke software. USPIO causes a rapid decline in T2*, which is the constant for the decay of MRI signal intensity over time, and as such, changes in T2* can be used to assess USPIO accumulation in the AAA [8]. Colour maps representing the percentage change in T2* (%∆T2*) were generated using custom written scripts (MATLAB, The MathWorks Inc., Natick, MA). In order to minimise the effect of artefact, a threshold of ≥ 71% ∆T2* was used to identify areas of signal change attributable to true USPIO uptake, based on previous reproducibility data from our group [16].

Colour maps were classified according to predefined criteria [8], into USPIO-negative (no mural USPIO uptake) or USPIO-positive (area(s) of significant mural USPIO enhancement indicative of macrophage-driven inflammation). Significant mural USPIO enhancement was defined as 10 or more contiguous USPIO-positive voxels adjacent to the aneurysm wall. Periluminal USPIO uptake is not considered to represent true inflammation and likely represents passive trapping of USPIOs in periluminal thrombus [17] although mural and periluminal uptake can be difficult to distinguish from posterior wall uptake due to their close proximity.

### Computed Tomography Three-Dimensional Reconstruction and Meshing

A standard high-resolution contrast-enhanced CTA was performed with a slice thickness of 1.0 mm and pixel size of 0.625 mm (Aquilion One, Toshiba Medical Systems Ltd., UK). These images were used to create patient-specific models of each aneurysm. Segmentation and 3D reconstruction were performed using commercial software (VASCOPS GmbH, Sweden). Early studies of AAA often assumed a uniform wall thickness [18]; however, variable wall thickness has been shown to highly influence the predicted results [12, 19–22]. Therefore, this package employs a specialist algorithm to calculate a more physiological aneurysm wall thickness distribution, which varies between 1.5 and 1.13 mm at the thrombus-free and covered sites, respectively [19]. Finite element (FE) meshes were then created from the 3D aneurysm geometry using the A4 clinical research software (VASCOPS). After suitable refinement, each AAA volume mesh typically consisted of > 160,000 (C3D8H) elements. These meshes were then exported to Abaqus 6.10-1 (Dassault Systemes, Simulia, Providence, RI, USA) for analysis. Both the aortic wall and thrombus regions were modelled as hyperelastic, homogeneous, incompressible, and isotropic materials, using well-established constitutive models [23, 24] with material constants based on population data. Loading representative of peak systolic blood pressure was applied as an outward-facing uniformly distributed pressure load acting on the luminal surface of the aneurysm. To remove any variability due to loading and to allow for comparison across patient cases, a peak systolic blood pressure of 120 mmHg (0.016 MPa) was chosen, as in many previous studies [25, 26]. In the present study, the effect of wall shear stress due to blood flow was not considered due to its negligible magnitude. Residual stresses in the aortic wall itself and the interaction of the aorta with the surrounding structures of the body (e.g. organs and spine) were also not considered. However, displacements at the distal and proximal most regions of each aneurysm were restrained, in all degrees of freedom, to model attachment of the AAA to the rest of the aorta.

For efficiency, a custom script was developed in Python (Python Software Foundation, Python Language Reference, version 2.7, available at http://www.python.org) to automate the definition of the model parameters and batch process all 50 patients. All simulations were computed on a Dell Precision T7600 workstation with 16 cores and 64 GB of RAM. Contour plots of von Mises stress were output for all aneurysms, and their locations were manually aligned to USPIO uptake colour maps. Maximum AAA diameter, as measured orthogonal to the AAA centreline, was also extracted from each CT reconstruction.

### Comparison of Two-Dimensional Contour Plots and USPIO Uptake on Colour Maps

**Fig. 2:**
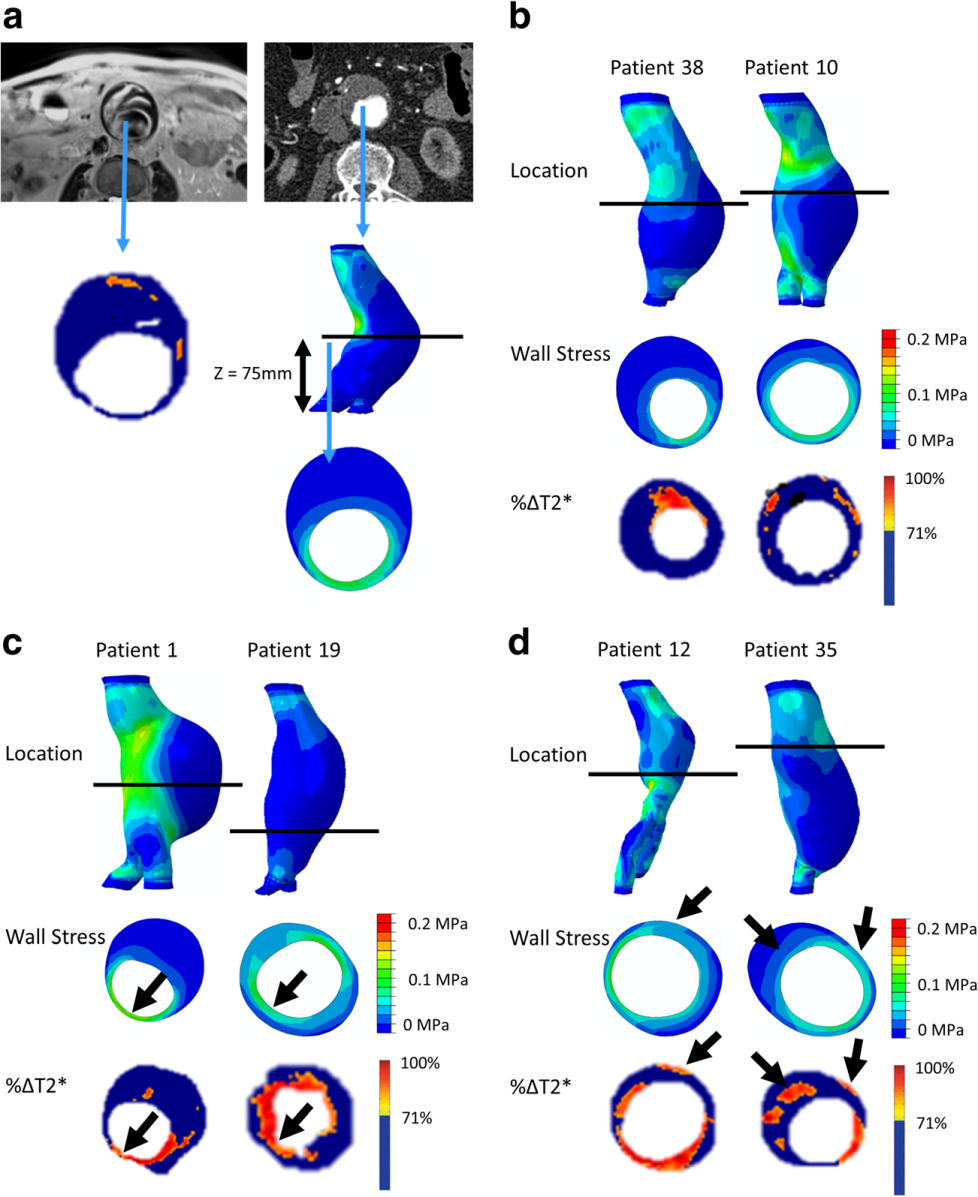
(**a**) The slice with the largest mural USPIO enhancement (most diseased segment) is selected from MRI. The corresponding slice is then extracted from the CT-based FE model using relevant anatomical landmarks (e.g. Z-distance from the iliac bifurcation). The contours are then visually compared to determine if co-location occurs. Examples of aneurysms with no co-location, some periluminal co-location (which does not represent inflammation), and true co-location of elevated stress with significant mural USPIO enhancement can be seen in panels (**b**), (**c**), and (**d**), respectively. Note: *black arrows* point to approximate regions of co-location

In order to compare the spatial relationship between elevated stress and areas of inflammation represented by USPIO uptake, the two-dimensional (2D) contour maps of von Mises stress were manually co-aligned with the USPIO colour maps. The MRI slice with the largest area of USPIO uptake (i.e. most diseased segment) was chosen for analysis, ensuring that the corresponding cross-sectional slice was analysed from the 2D contour map (Fig. 2a). Regions of elevated stress and areas of mural USPIO enhancement were examined for visual overlap on the chosen slice.

### Global Comparisons: Whole AAA Analysis

Global comparisons between peak wall stress (PWS) predicted for each patient (from FE) and maximum and peak USPIO uptake (%∆T2*) per patient were also investigated, using values derived from the entire aneurysm. Maximum AAA diameter was also included in the analysis, as this is the most widely used predictor of aneurysm rupture. To test if correlations varied with classification of USPIO uptake (i.e. USPIO-negative or USPIO-positive), comparisons by group were also investigated.

### Mural USPIO Enhancement

To examine the trends with respect to the focal mural inflammation observed in USPIO-positive aneurysms more closely, the correlation of diameter and whole-vessel PWS with mural USPIO uptake values (mean and peak USPIO values identified on the most diseased segment) was investigated. Non-focal areas of USPIO uptake (i.e. those which did not meet the definition of mural USPIO enhancement) were removed prior to this analysis.

### Statistical Analysis

Linear dependence between variables was investigated using the Pearson’s correlation coefficient. Unpaired two-sample *t* tests were used to assess differences in mean values between AAA groups. Statistical significance was determined using a two-tailed *p* < 0.05. All statistical analyses were performed using Minitab® 17 (Minitab Ltd., Coventry CV 2TE, UK).

## Results

Fifty patients were included in this study who were predominantly elderly (mean age of 72 (65–87) years) men (90%), with a mean CT AAA diameter of 52.96 (40.60–69.40) mm. Mural USPIO enhancement was seen in 21 (42%) patients who had a significantly greater mean AAA diameter (55.19 vs 51.34 mm without mural enhancement, *p* = 0.0255). There was no difference between groups in terms of other relevant characteristics (Table 1).

**Table 1:** Summary demographics of patients, per USPIO classification group, using comparisons of proportion or chi-squared test, where appropriate

Variable	All patients (*n* = 50)	USPIO-negative (*n* = 29)	USPIO-positive (*n* = 21)	Difference between groups—*p* value
Age in years (SD)	72.34 (6.32)	71.96 (6.37)	72.86 (6.37)	0.6372
Male sex (%)	45 (90)	25 (86)	20 (95)	0.383
Mean AAA diameter in mm (SD)	52.96 (6.08)	51.34 (5.19)	55.19 (6.62)	0.0255
Past medical history				
Family history of AAA (%)	12 (24)	6 (20.69)	6 (28.57)	0.738
Coronary artery disease (%)	16 (32)	9 (31.03)	7 (33.33)	1
Stroke or transient ischemic attack (%)	3 (6)	2 (6.90)	1 (4.76)	1
Peripheral vascular disease (%)	3 (6)	3 (10.34)	0 (0)	0.254
Cerebrovascular disease (%)	2 (4)	2 (6.90)	0 (0)	0.503
Risk factors				
Current smoking habit (%)	12 (24)	5 (17.24)	7 (33.33)	0.314
Previous smoking habit (%)	32 (64)	20 (68.97)	12 (57.14)	0.551
Hypertension (%)	40 (80)	24 (82.76)	16 (76.20)	0.7232
Hypercholesterolaemia (%)	44 (88)	25 (86.21)	19 (90.48)	1
Diabetes (%)	7 (14)	4 (13.79)	3 (14.29)	1
Medication				
Anti-diabetes medication (%)	6 (12)	5 (17.24)	1 (4.76)	0.38
Statin therapy (%)	2 (4)	2 (6.90)	0 (0)	0.503
Anti-coagulant therapy (%)	1 (2)	0 (0)	1 (4.76)	0.42

### 2D Contour Plot and USPIO Colour Map Comparison

The maximum areas of PWS were most commonly (*n* = 28; 56%) found in the posterior wall and regions of high curvature such as the shoulder region. When analysing the most diseased segment, 40 aneurysms (80%) demonstrated some degree of visual overlap between regions of elevated stress and USPIO enhancement anywhere (e.g. adjacent to lumen and/or wall) in the vessel, while in the remaining 10 cases, no visual overlap was observed (e.g. Fig. 2b). Of the 40 aneurysms which did exhibit overlap, only 19 (38%) demonstrated spatial co-location of increased stress and USPIO enhancement adjacent to the aneurysm wall, while the remaining aneurysms exhibited overlap of elevated stress at areas of peri-luminal USPIO uptake where classification is challenging (e.g. Fig. 2c). Overall, only eight aneurysms (16%) demonstrated co-location of elevated stress with an area meeting the definition of mural USPIO enhancement (e.g. Fig. 2d).

Details of inter- and intra-observer variability for stress and USPIO comparisons as well as additional contour plot comparisons for the full 50 patients can be found in the supplementary text (ESM 1).

### Global Comparisons: Whole AAA Analysis

The average PWS for all aneurysms was 0.1980 MPa. There was no difference between the average PWS for USPIO-negative aneurysms (0.1999 ± 0.1326 MPa) and USPIO-positive aneurysms (0.1955 ± 0.1495 MPa; *p* = 0.83, 95% CI −0.0920 to 0.0740).

**Fig. 3:**
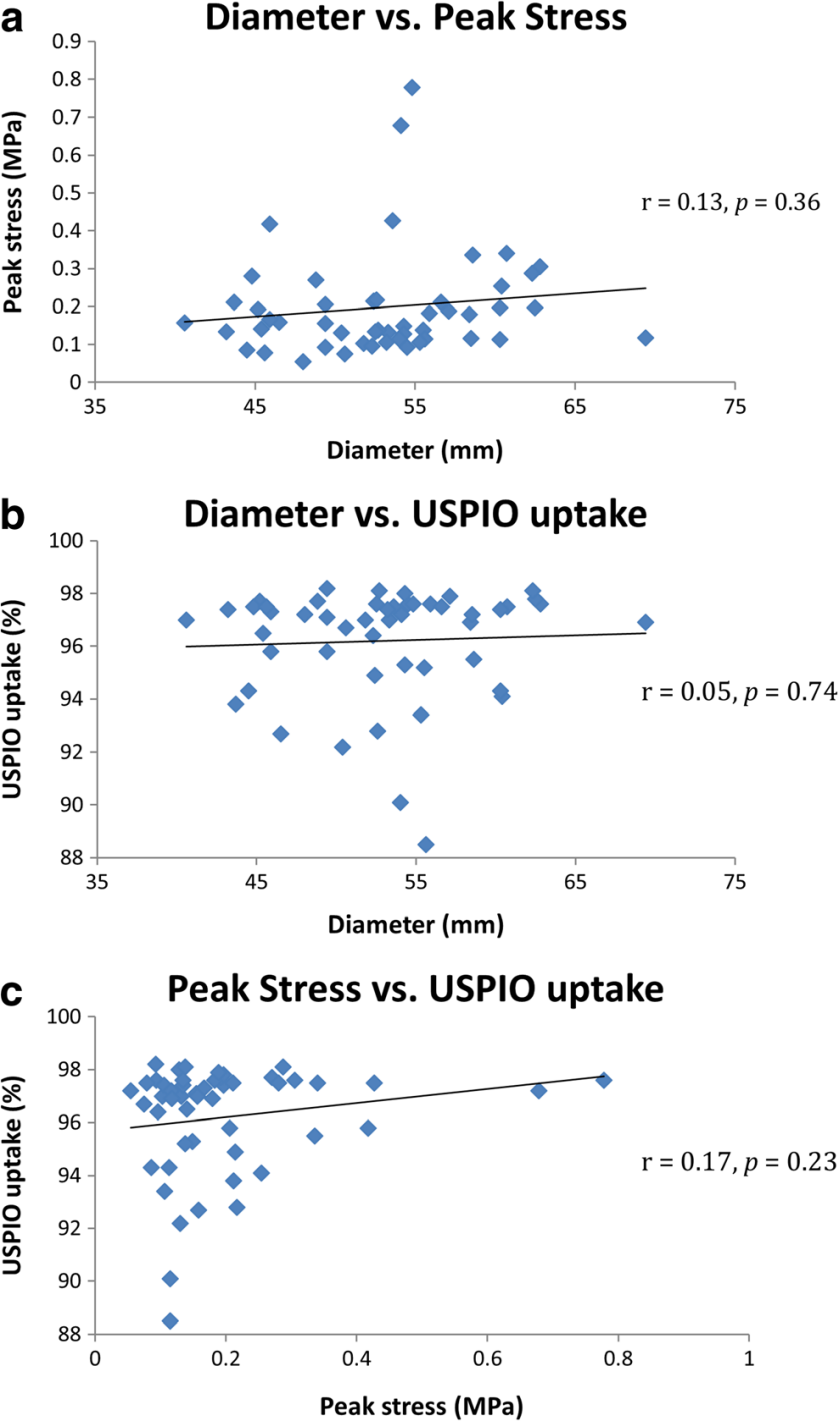
Global comparisons (*n* = 50) of aneurysm diameter, peak stress predicted by finite element analysis, and % ∆T2* USPIO, using data from the entire aneurysm. There are no significant correlations between any of the measured parameters

Maximum diameter was not associated with PWS (*r* = 0.13, *p* = 0.36) or maximum USPIO uptake (*r* = 0.05, *p* = 0.74). There was no correlation between PWS and maximum USPIO enhancement over the entire aneurysm (*r* = 0.17, *p* = 0.23), as shown in Fig. 3.

**Fig. 4:**
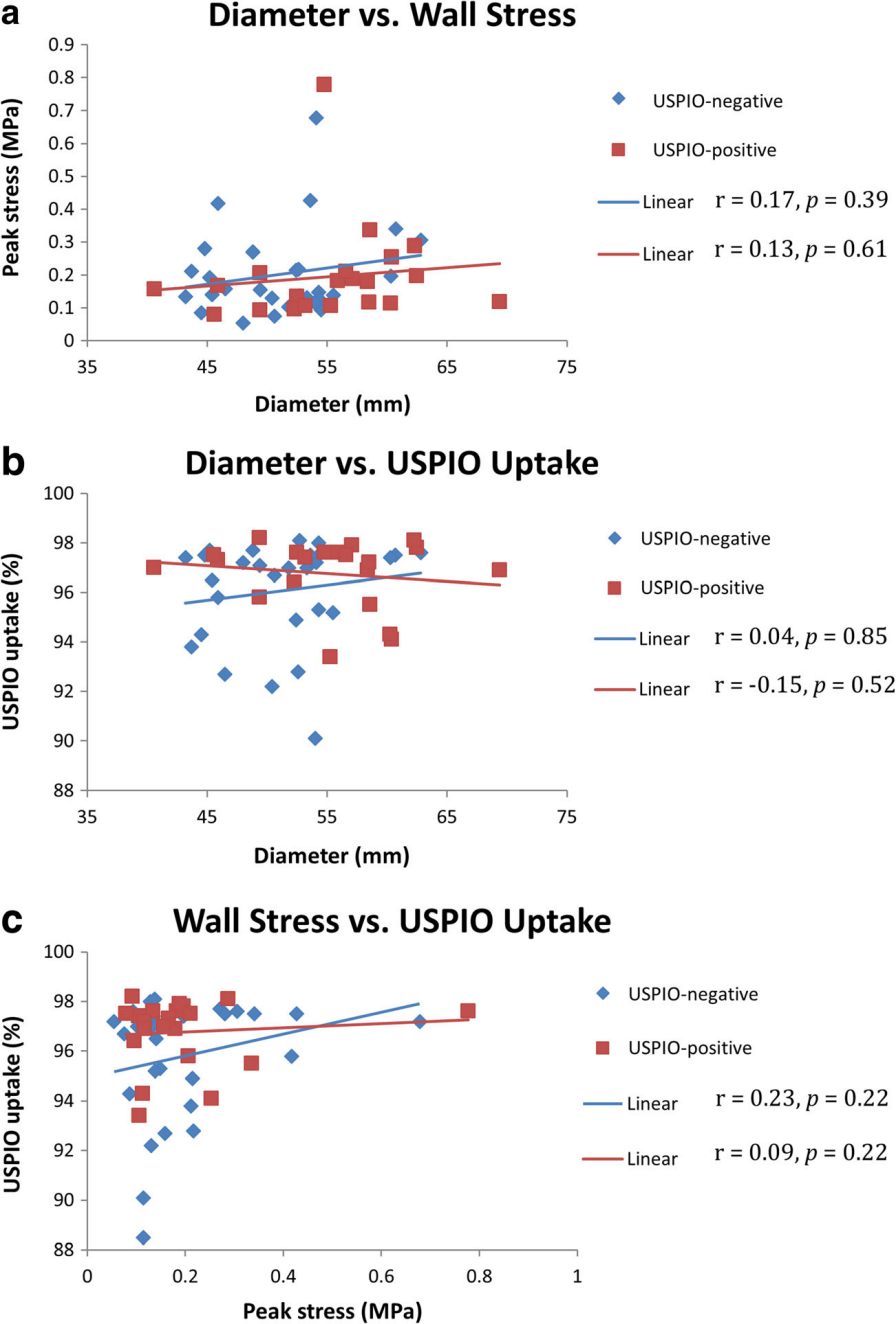
Comparisons of diameter, peak stress, and % ∆T2* USPIO uptake per group; USPIO-negative (*n* = 29) vs USPIO-positive (*n* = 21), using data from the entire aneurysm. No significant correlations demonstrated

When comparing between groups, diameter was not correlated with PWS in either group (USPIO-negative *r* = 0.17, *p* = 0.39; USPIO-positive *r* = 0.13, *p* = 0.61), nor was diameter and peak USPIO uptake (USPIO-negative *r* = 0.04, *p* = 0.85; USPIO-positive *r* = −0.15, *p* = 0.52), as shown in Fig. 4. There was no difference in correlation between PWS and mural USPIO enhancement between groups (*r* = 0.23 vs. *r* = 0.09; *p* = 0.22).

### Focal Mural USPIO Enhancement

**Fig. 5:**
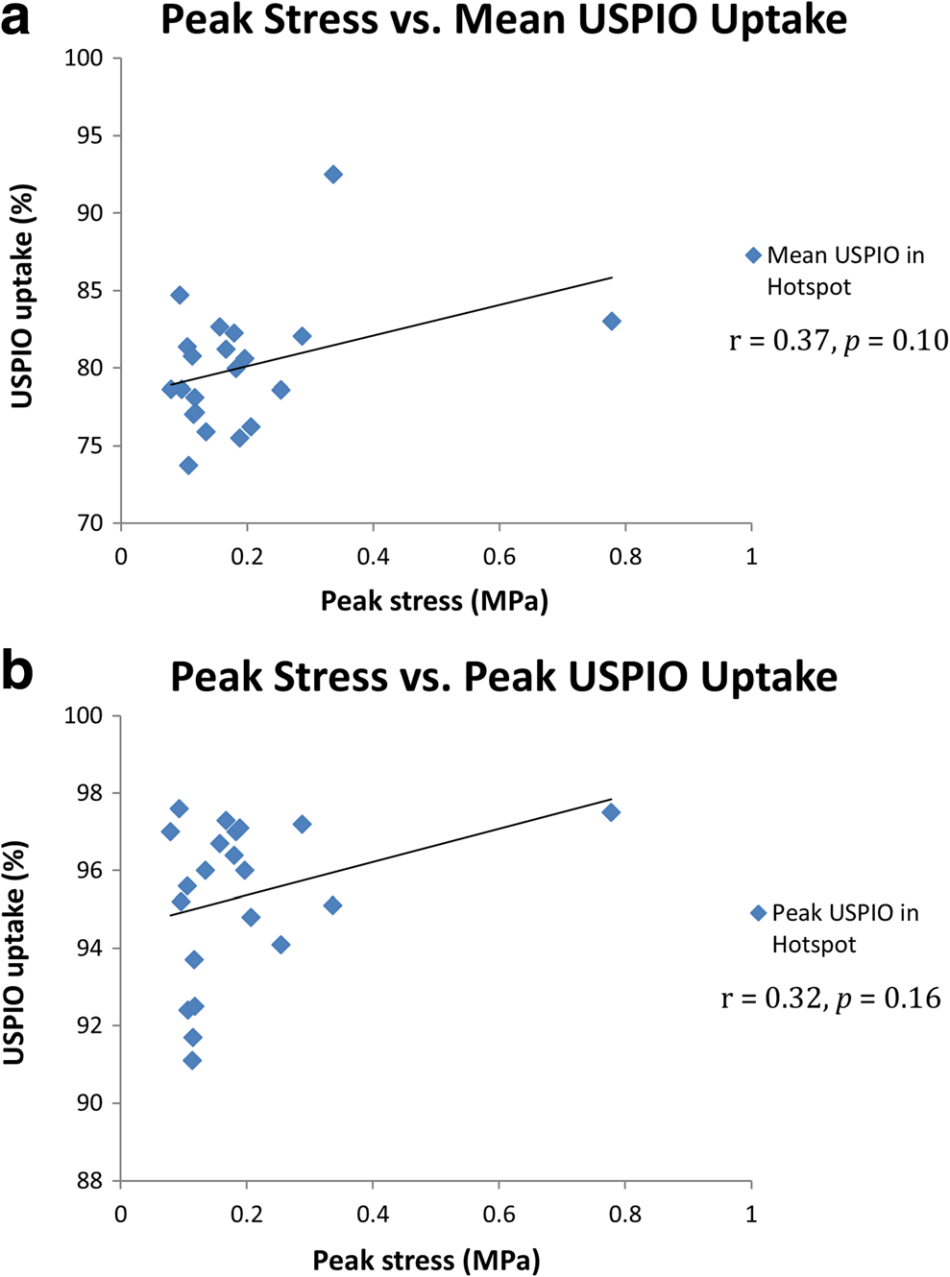
Mural USPIO enhancement analysis. Correlation between peak stress (derived from the entire aneurysm) with mean and peak USPIO uptake on the most diseased segment (*n* = 21). No significant correlations identified

There were no associations between PWS and mean or peak USPIO uptake within the individual areas of mural USPIO enhancement on the most diseased segment (*r* = 0.37, *p* = 0.10; *r* = 0.32, *p* = 0.16), as shown in Fig. 5a and b respectively.

**Fig. 6:**
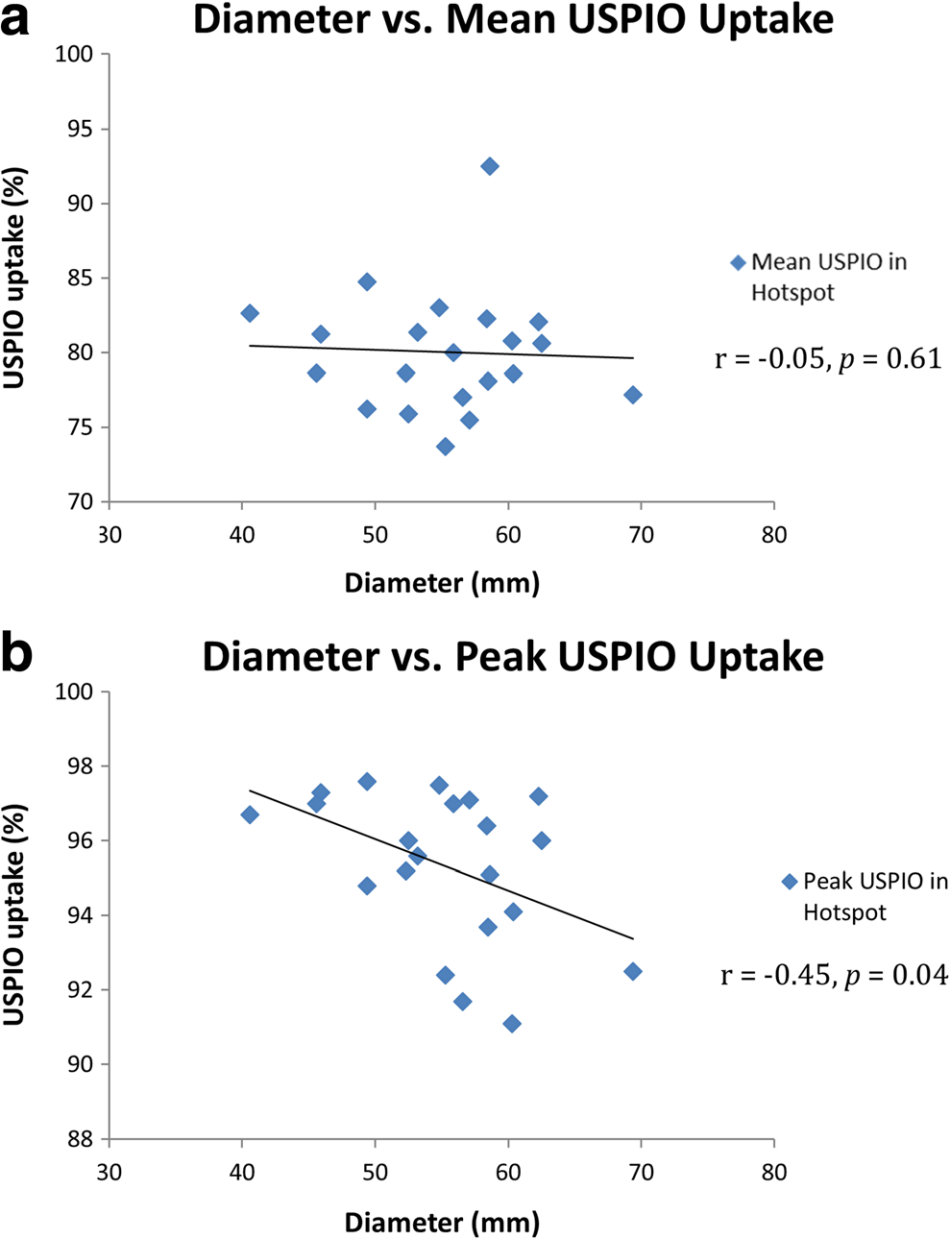
Mural USPIO enhancement analysis. Correlation between aneurysm diameter with mean and peak USPIO uptake in the identified areas of significant mural USPIO enhancement on the most diseased segment (*n* = 21)

Similarly, mean USPIO uptake within the identified regions of mural USPIO enhancement did not correlate with diameter (*r* = −0.05, *p* = 0.61), as shown in Fig. 6a. However, a significant weak inverse correlation was observed between peak USPIO uptake and diameter (*r* = −0.45, *p* = 0.04), as shown in Fig. 6b.

## Discussion

In the present study, we analysed the spatial relationship between two biomarkers of AAA rupture risk; namely, inflammation detected through the uptake of USPIO and wall stress calculated using patient-specific FE models. Elevated stress was commonly observed in areas vulnerable to rupture (e.g. the posterior wall and shoulder), while regions of USPIO enhancement typically occurred near the wall behind thick thrombus. Only 16% of aneurysms exhibited co-localisation of elevated stress and mural USPIO enhancement. Globally, no correlation was observed between peak stress and other measures of USPIO uptake, e.g. mean or peak uptake.

The maximal area of PWS (as demonstrated using FE modelling) is most commonly found in the posterior aneurysm wall, where thrombus is typically thin or absent and the blood pool is in close proximity. Up to 82% of AAA ruptures occur on the posterior wall [27], yet most aneurysms bulge anteriorly. This may be explained by the restriction in radial expansion of the posterior wall due to the spinal column, with increasing anterior asymmetry resulting in an increase in posterior wall peak stress [28]. In addition, PWS was also frequently observed in regions of high curvature, including the shoulder region. These findings have been reported in many previous FE studies [29, 30], some of which have demonstrated that increased stress at the aneurysm shoulder is associated with AAA expansion [11], possibly due to stress-induced changes in vessel wall stiffness [3].

Despite there being general visual overlap between stress and USPIO uptake, only 16% of all aneurysms in our study demonstrated co-location of elevated stress with mural USPIO enhancement. The finding of co-location of elevated stress and USPIO uptake in the posterior wall is challenging because this is a very thin structure and we cannot resolve whether this represents mural or periluminal uptake of USPIOs. Periluminal USPIO uptake is non-specific and reflects the passive trapping of USPIOs within the gelatinous thrombus immediately adjacent to the lumen. Histological analysis of aneurysm tissue has demonstrated that the immediate luminal aspect consists of freshly deposited thrombus that is highly cellular (including macrophages) with progressive organisation towards the abluminal surface, which is acellular and consists largely of fibrin [31]. Therefore, the presence of USPIO uptake in the fibrinous thrombus adjacent to the aneurysm wall can be considered to represent true inflammation, whereas periluminal uptake of USPIO does not represent true cellular inflammation. Gadolinium-based MRI studies appear to reiterate this—the luminal surface of thrombus enhances with gadolinium, in contrast to the organised part of the thrombus and the aortic wall, which typically do not [8].

With regards to co-location of PWS and mean, maximum, or peak USPIO uptake, no significant associations were demonstrated. These findings suggest that areas of elevated stress and objective measures of focal inflammation (as demonstrated on USPIO-enhanced MRI) do not commonly co-locate in AAA disease. While we know that focal mechanical and biological processes do contribute towards AAA disease progression and rupture, it appears that stress and inflammation represent two distinct processes that are not necessarily connected spatially or causally. Our previous pilot study of USPIO MRI demonstrated a link between regions of focal cellular inflammation and future AAA expansion. Indeed, histological analysis of tissue obtained during AAA repair provided evidence of the accumulation of USPIO within macrophages, and USPIO-positive aneurysms were found to expand three times more rapidly than USPIO-negative aneurysms [8].

Other molecular imaging studies of AAA wall inflammation using positron emission tomography (PET) have demonstrated the ability of ^18^F-FDG to identify regions of inflammation, confirmed by histological analysis [32]. However, there is no definite consensus as to whether increased ^18^F-FDG uptake correlates with clinical outcomes such as expansion or rupture [33, 34]. In addition, we recently examined vascular inflammation in a small population of AAAs (*n* = 15) and found some but not a close correlation between ^18^F-FDG uptake on PET/CT and USPIO uptake on MRI [17]. This may reflect the different elements of macrophage activity that these imaging techniques detect: glycolysis and phagocytosis, respectively.

Some other studies have also attempted to quantify the interaction between inflammation and peak stress, using a combined mechanical and biological approach to assess the overall stability of individual AAAs. In a pilot study (*n* = 5), Xu et al. reported a tentative link between ^18^F-FDG metabolic activity and high wall stress [30]. Later work by Maier et al. confirmed that wall stress predicted by FE and ^18^F-FDG uptake evaluated by PET/CT correlated both quantitatively and spatially in the cases examined (*n* = 18) [29]. Interestingly, our study has observed that high stress often occurs in regions of high curvature or inflection points, such as the aneurysm shoulder and posterior luminal surface, which have been reported to have high ^18^F-FDG uptake [6, 17, 29, 30]. However, more recent work by Nchimi and colleagues [6] on a larger sample size (*n* = 53) concluded that the relationship between ^18^F-FDG uptake and peak stress was not directly correlated, instead pointing to a complex multi-factorial relationship between increased ^18^F-FDG uptake and patient-specific factors such as aneurysm location (thoracic or abdominal), wall stress, and family history/patient lifestyle. The present study also suggests that the relationship between inflammation and peak stress is complex and potentially independent.

## Translational Impact and Clinical Implications

While each marker (e.g. USPIO uptake or stress) provides an independent indicator of aneurysm rupture risk, neither presents the full picture in isolation. However, when a combined approach such as presented in this study is taken, then added insight into the mechanical and biological conditions of the wall can be provided where indicators from these two independent markers coincide. Such insights may potentially lead to new methods for patient risk stratification, in particular in patients below the standard 55 mm intervention threshold or in larger aneurysms where the benefit/risk of intervention is less certain.

## Limitations and Future Direction

Our study has some technical limitations. We employed a continuum modelling strategy for the intraluminal thrombus, which is thought to act as a mechanical buffer [35], meaning that stress behind thick thrombus may be under-represented. Improvements in thrombus modelling may improve this [36]; however, our strategy is in keeping with most previous studies. In addition, our regions of mural USPIO enhancement were identified on axial slices in the 2D plane—this is based on the techniques used in our previous pilot study and is considered a reasonable, pragmatic approach to image analysis. However, our group is also exploring 3D mural USPIO enhancement detection [37], which may provide additional data with which to compare wall stress in the future. Furthermore, as in many previous studies, we have assumed population-mean parameters for the AAA material properties, which may influence the resulting wall stress. Ongoing work aims to eliminate the potential uncertainty surrounding material properties [38], and it is hoped that this step in combination with a more comprehensive comparison of USPIO-uptake and PWS in 3D may provide some further insight into the complex interplay of these two factors in AAA disease progression. One final limitation is the lack of longitudinal data with which to verify our hypothesis that overlapping regions of stress and USPIO may potentially identify patients at greater risk of rupture. Recent findings from the main MA^3^RS study [39] have already demonstrated that USPIO uptake predicts expansion, but that this is not independent of diameter. Final follow-up data from the trial has been collected, and we have recently started to explore other secondary analyses, including how baseline stress/USPIO uptake predictions relate to the evolution of the aneurysm over time. However, given the non-trivial nature of this work, it will take many months before the data is fully analysed and interpreted. Once analysed, it is anticipated that these findings will provide a greater level of insight into disease progression and the significance of the overlapping regions of stress and inflammation.

## Conclusions

In this combined clinical and FE study of 50 aneurysms, poor correlations between USPIO uptake and stress suggest that these biological and mechanical factors address different aspects of the aberrant pathway towards disease progression in AAA. We observed that peak stress most commonly occurs in regions of increased curvature (such as the posterior wall and inflection points), and further correlation with peak stress and different markers of inflammation is warranted. While both macrophage-mediated inflammation and peak wall stress play a part in AAA expansion and rupture, they do not spatially co-locate, and this only serves to reinforce the complex multi-factorial elements in aneurysm disease progression. However, it remains a possibility that although PWS and mural USPIO enhancement are independent processes, when they co-localise, this could be a trigger for aneurysm rupture. To address this hypothesis requires long-term follow-up of clinical cohorts. Additional clinical and biomechanical studies are therefore required to further investigate the synergy between biological and biomechanical aspects of AAA disease.

## Electronic supplementary material

ESM 1 (DOCX 5861 kb)
