# Supplementary material for: Exploring the Biological and Mechanical Properties of Abdominal Aortic Aneurysms Using USPIO MRI and Peak Tissue Stress: A Combined Clinical and Finite Element Study
Source: J Cardiovasc Transl Res. 2017 Aug 14;10(5):489–98. doi: 10.1007/s12265-017-9766-9 (PMC5722953; doi:10.1007/s12265-017-9766-9)
Supplement: Supplementary file 1 — (DOCX 5861 kb) [file 12265_2017_9766_MOESM1_ESM.docx]

**Exploring the biological and mechanical properties of abdominal aortic aneurysms using USPIO MRI and peak tissue stress:**

**A combined clinical and finite element study**

**Journal name:** Journal of Cardiovascular Translational Research

**Authors:**

Noel Conlisk^1,2,7^, Rachael O. Forsythe^1,2,3^, Lyam Hollis^1^, Barry J. Doyle^1,5,6^, Olivia M.B. McBride^1,2,3^, Jennifer M.J. Robson^1,2^, Chengjia Wang^1,3^, Calum D. Gray^3^, Scott I.K. Semple^1,3^, Tom MacGillivray^1,4^, Edwin J.R. van Beek^1,3^, David E. Newby**^1^**^,3^, Peter R. Hoskins^1,7^

^1^Centre for Cardiovascular Science, The University of Edinburgh, Edinburgh, UK

^2^School of Clinical Sciences, The University of Edinburgh, Edinburgh, UK

^3^Clinical Research Imaging Centre, The University of Edinburgh, Edinburgh, UK

^4^Centre for Clinical Brain Sciences, The University of Edinburgh, Edinburgh, UK

^5^Vascular Engineering Laboratory, Harry Perkins Institute of Medical Research, Perth, Australia

^6^School of Mechanical and Chemical Engineering, The University of Western Australia, Perth, Australia

^7^Institute for Bioengineering, The University of Edinburgh, Edinburgh, UK

**Corresponding author:**

Dr. Noel Conlisk

Institute for Bioengineering

The University of Edinburgh

Faraday Building

The King's Buildings

Mayfield Road

Edinburgh

EH9 3JL

Phone: +44 (0) 7775 332506

Fax: +44(0) 131 242 9101

Email: [noel.conlisk@ed.ac.uk](mailto:noel.conlisk@ed.ac.uk)

**Supplement:**

The first section of this supplement contains information on the inter- and intra-observer variability for both the two-dimensional stress and USPIO comparison method and the classification of inflammation from USPIO MRI scans. The second section of this supplement contains the contour plots and colour map comparisons of wall stress and USPIO uptake for all 50 patients (Fig. 7 – 11).

*Inter- and intra-observer variability*

Inter- and intra-observer variability for stress and USPIO comparisons was analysed in a sub-set of 20 patients. Two trained observers independently evaluated the co-location of inflammation and stress, blinded to previous results. When analyzing co-location at the periluminal area, the proportional agreement was 0.95, kappa 0.773, representing substantial agreement. When examining co-location of stress and USPIO uptake at the aneurysm wall, the proportional agreement was 0.85, kappa 0.659, representing substantial agreement. The first observer repeated the analysis at an interval of 6 months, blinded to previous results; for peri-luminal co-location, the proportional agreement was 1.0, kappa 1.0, representing perfect agreement, and for aneurysm wall co-location, the proportional agreement was 0.85, kappa 0.681, representing substantial agreement. Therefore the present method of analysis demonstrates very good observer agreement.

Similarly, the proportional intra-observer agreement for the classification of inflammation from USPIO MRI scans was 0.91, with a kappa value of 0.82. When two trained observers independently classified the USPIO MRI scans, the proportional inter-observer agreement was 0.83, kappa 0.66.

*2D contour plot and USPIO colour map comparisons*


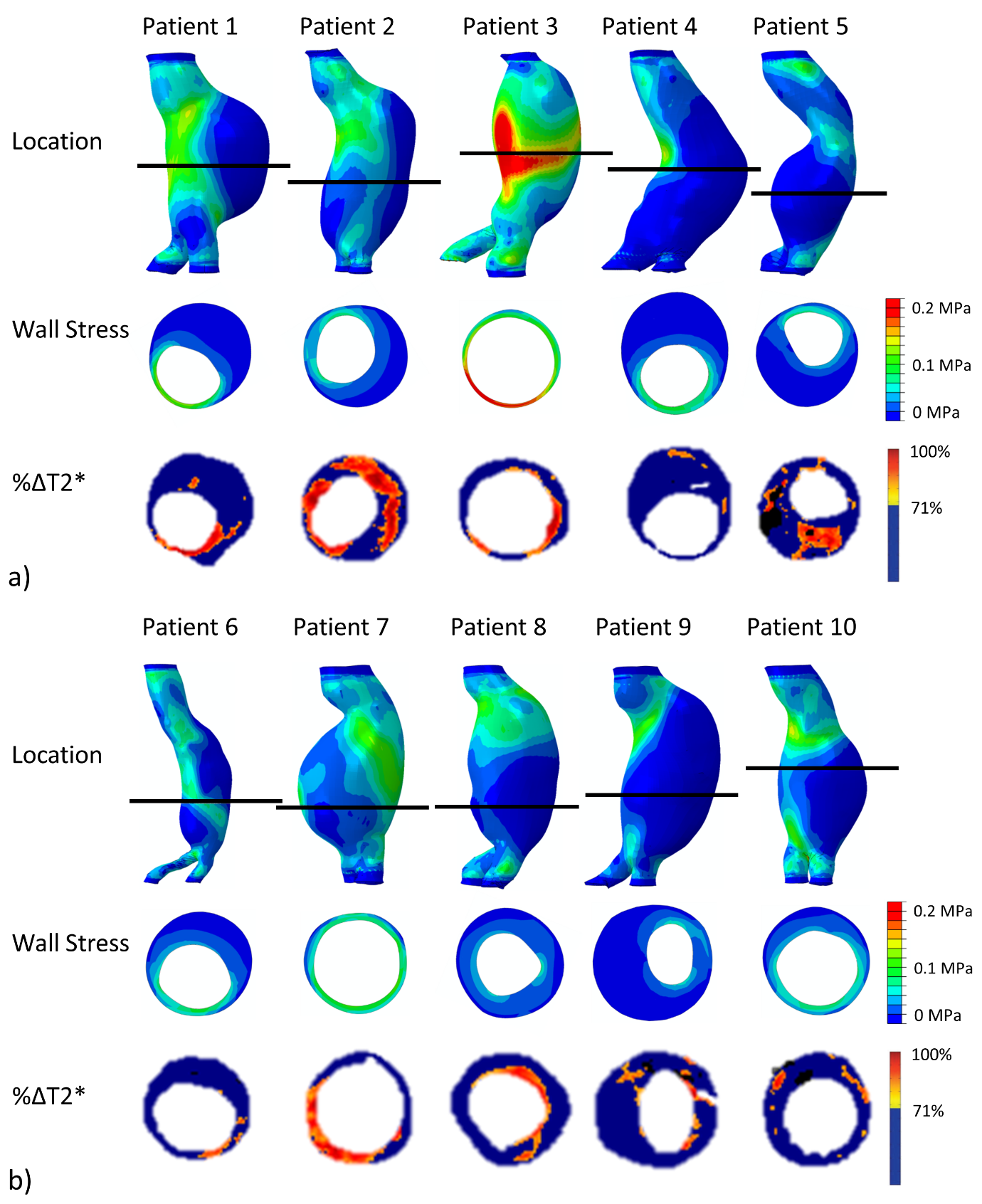


**Fig. 7** Comparison of wall stress and USPIO uptake in patients 1 - 10.


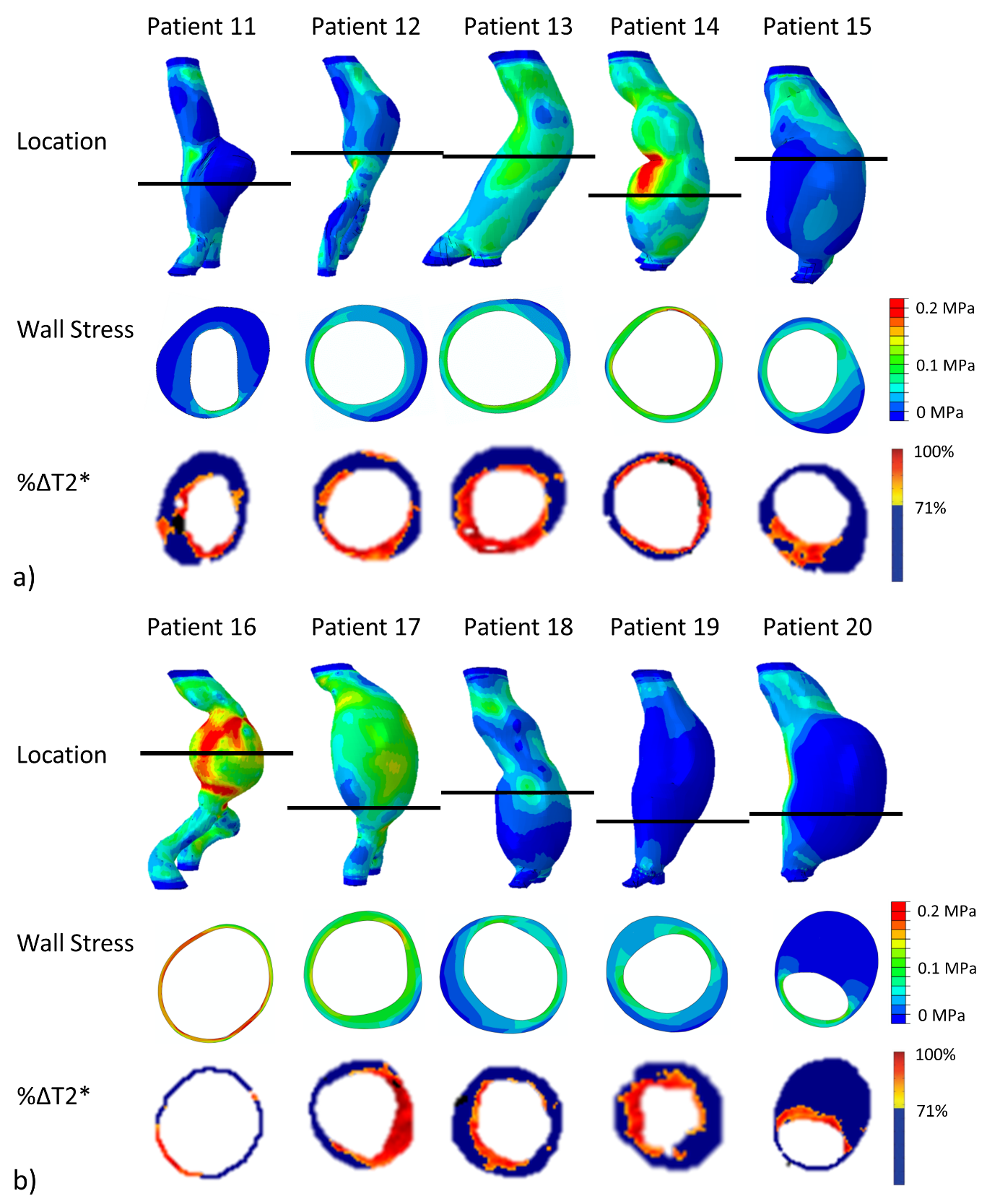


**Fig. 8** Comparison of wall stress and USPIO uptake in patients 11 - 20.


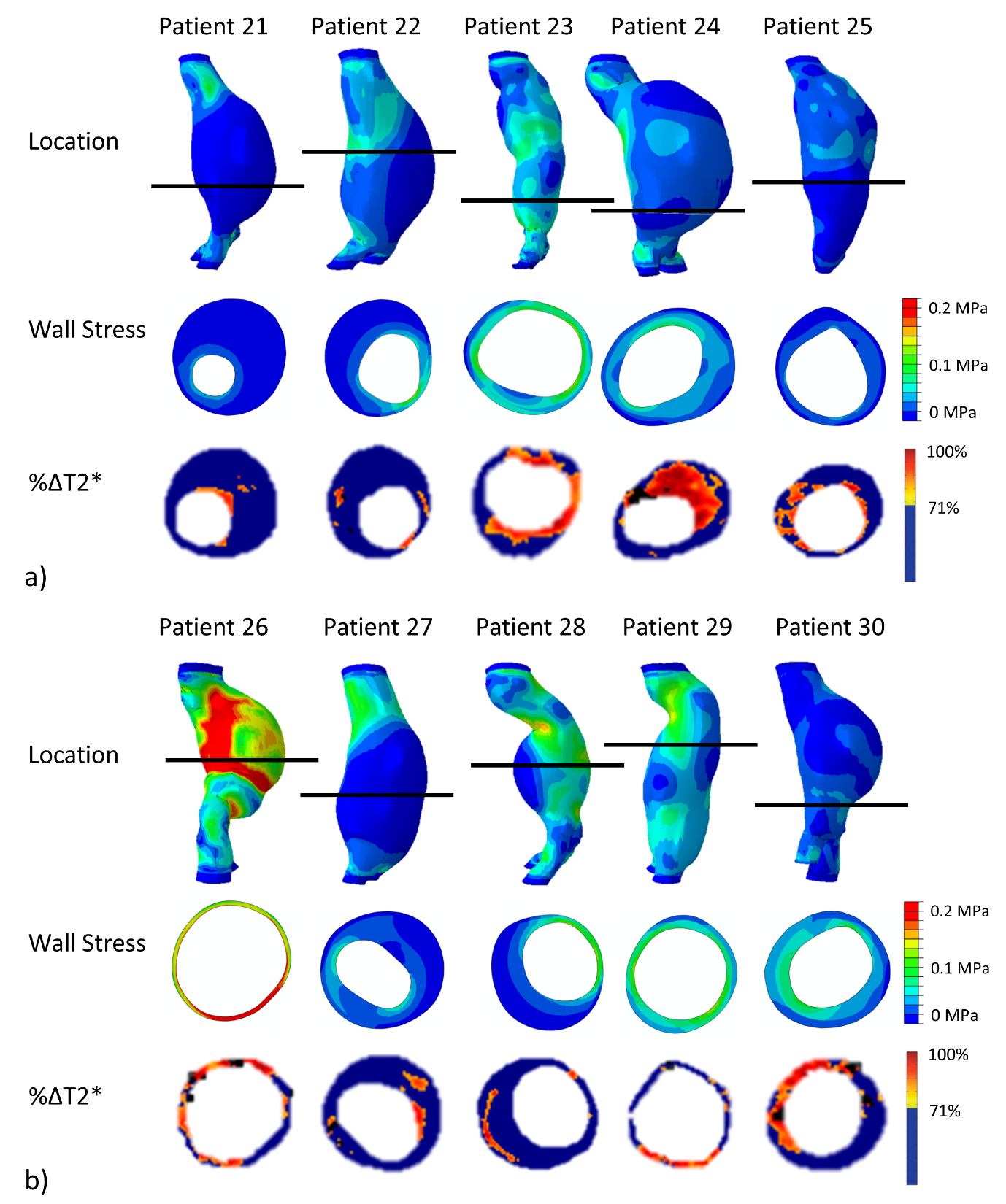


**Fig. 9** Comparison of wall stress and USPIO uptake in patients 21 - 30.


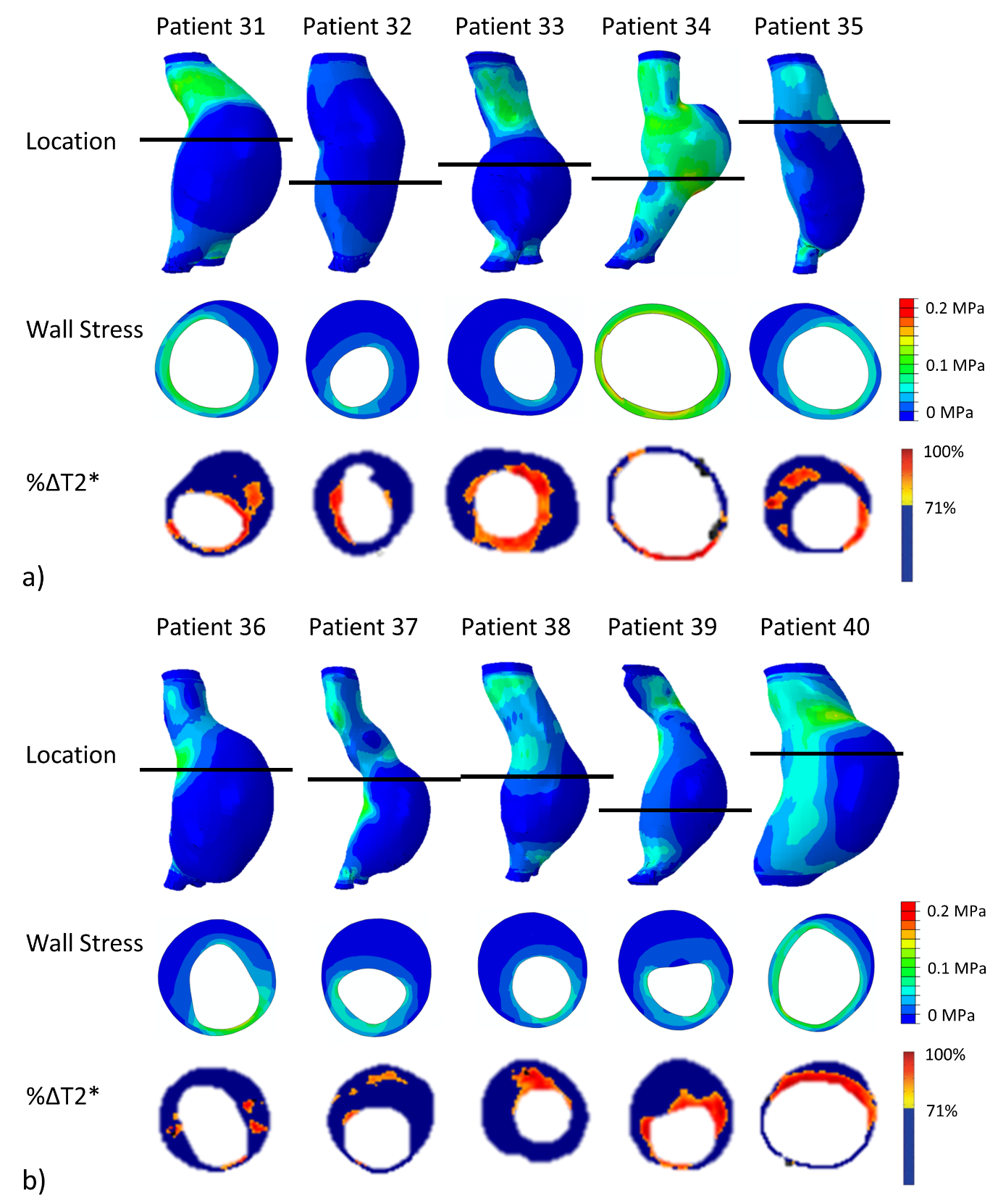


**Fig. 10** Comparison of wall stress and USPIO uptake in patients 31 - 40.
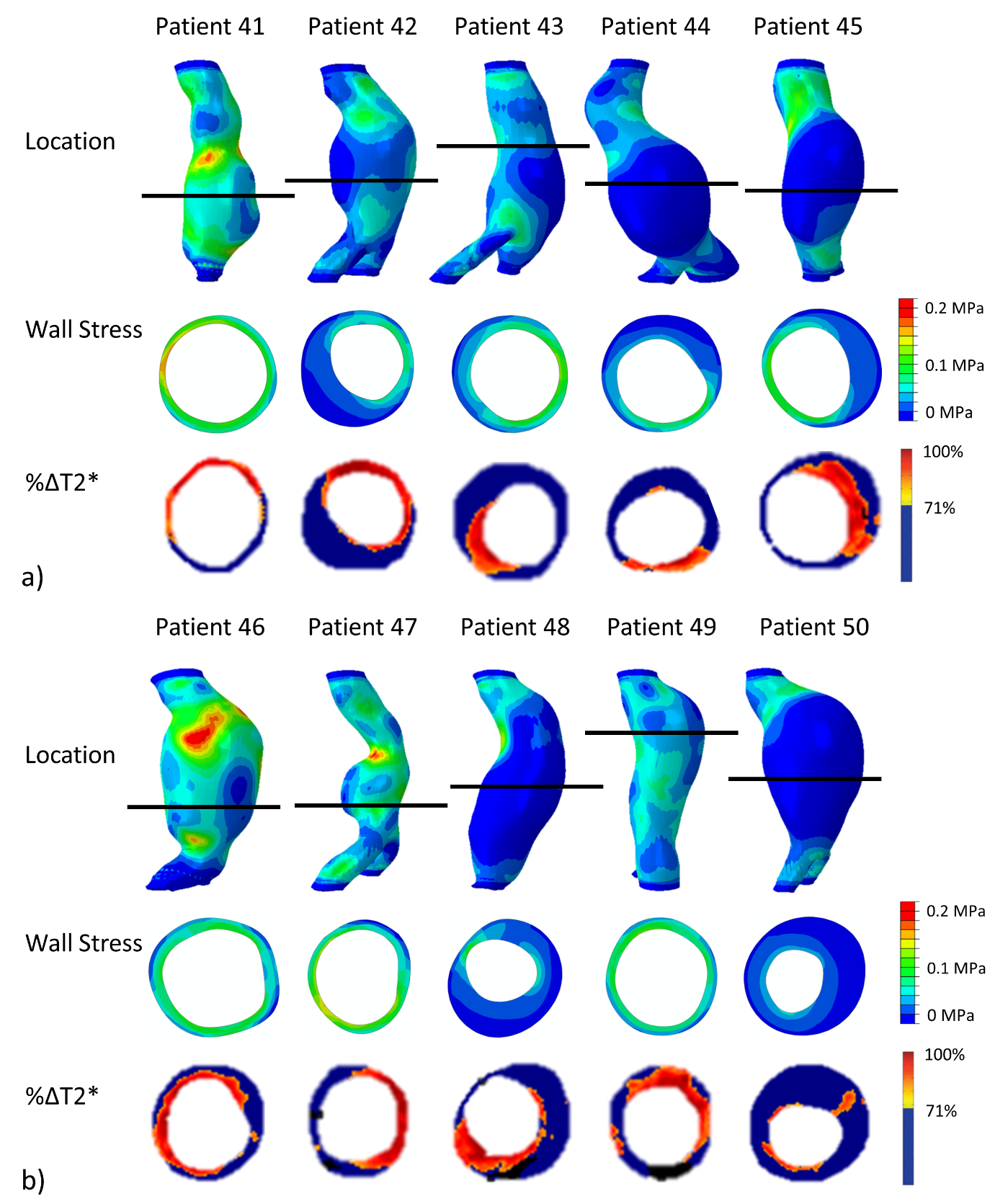


**Fig. 11** Comparison of wall stress and USPIO uptake in patients 41 - 50.
